# Hsa-miR-330-5p Aggravates Thyroid Carcinoma via Targeting FOXE1

**DOI:** 10.1155/2021/1070365

**Published:** 2021-07-02

**Authors:** Yuxia Wang, Zhaozhe Liu, Xiangning Ren, Nan Sun, Qiuhua Li, Che Bian

**Affiliations:** ^1^Department of Endocrinology, The Fourth Affiliated Hospital of China Medical University, Shenyang 110032, Liaoning, China; ^2^Oncology Department, General Hospital of Northern Theater Command, Shenyang 110014, Liaoning, China; ^3^Oncology Department, The Second Affiliated Hospital of Liaoning University of Traditional Chinese Medicine, Shenyang 110034, Liaoning, China

## Abstract

**Background:**

Thyroid carcinoma (TC) is one of the frequent endocrine malignancies, and growing evidence suggests that aberrant microRNA (miRNA) expression contributes to TC development and progression. Nevertheless, the function of miR-330-5p in the progression of TC remains unknown.

**Methods:**

The expression levels of miR-330-5 in patients with thyroid carcinoma and healthy controls were detected, and their potential diagnostic and prognostic values were analyzed.

**Results:**

In this study, we firstly found that miR-330-5p expression was markedly upregulated in TC tissue and cell lines. Functionally, the downregulation of miR-330-5p suppressed TC cell proliferation, migration, and invasion. Further studies revealed that miR-330-5p negatively regulated the expression of forkhead box E1 (FOXE1). More importantly, the results of rescue experiments suggested that FOXE1 overexpression reduced the positive effects of miR-330-5p overexpression in TPC-1 and K-1 cells.

**Conclusions:**

This work revealed that miR-330-5p facilitated the TC progression through targeting FOXE1, which may offer novel therapeutic options for TC.

## 1. Introduction

Thyroid carcinoma (TC) is considered to be the most common endocrine malignancy, accounting for nearly one-third of head and neck malignancies in the world [1]. The conventional treatment strategies, including thyroidectomy, thyrotropin suppression therapy, and radioactive iodine (RAI) ablation, provide advantageous treatment outcomes in the majority of TC cases [2, 3]. However, the clinical outcome of advanced TC is far from satisfactory. Therefore, in order to unveil new and effective treatments, the molecular mechanism underlying TC needs to be clarified.

MicroRNAs (miRNAs) are small noncoding RNAs that affect the downstream target gene expression at the posttranscriptional level and play crucial roles in regulating several biological processes including cell proliferation, apoptosis, and metastasis [4–6]. Growing lines of evidence have suggested that miRNAs play essential roles in the progression of TC [7–9]. For example, miRNA-15a regulates the proliferation and apoptosis of papillary thyroid carcinoma via regulating AKT pathway [10]. miR-429 suppresses cell growth and induces apoptosis of thyroid cancer cell by targeting ZEB1 [11]. On the other hand, miR-330 inhibits growth and migration of melanoma A375 cells [12]. A recent study also showed that miR-330-5p targets SPRY2 to promote hepatocellular carcinoma progression via MAPK/ERK signaling [13]. miR-330-5p suppresses glioblastoma cell proliferation and invasiveness through targeting ITGA5 [14]. However, the function and regulatory mechanism of miR-330-5p in TC remain unclear.

Forkhead box E1 (FOXE1) is an important member of the FOX transcription factor family. Increasing lines of evidence from genetic studies have shown that numerous genes in this family play vital roles in the process of tumor development [15, 16]. FOXE1 is a thyroid-specific transcription factor and plays fundamental roles in the growth of thyroid cells. Recently, its expression was found to be significantly higher in papillary thyroid carcinoma cell lines than in normal cell lines [17]. However, the regulatory effects between miR-330-5p and FOXE1 have not been documented in TC before.

In our study, we analyzed the role of miR-330-5p in the progression of TC. Our results indicated that the expression level of miR-330-5p was elevated in TC tissues and cell lines. Functionally, we confirmed that miR-330-5p promoted tumor proliferation, migration, and invasion. Further study revealed that miR-330-5p aggravated TC via targeting FOXE1. These findings indicated that miR-330-5p played a key role in the progression of TC.

## 2. Materials and Methods

### 2.1. Sample Collection

TC tissues and normal tissues were collected from 30 pathologically confirmed TC patients who underwent surgical treatment in General Hospital of Northern Theater Command. Those tissues were immediately frozen in liquid nitrogen after surgery and stored at −80°C. All patients did not get any anticancer treatment before surgery. Each patient had signed a written informed consent. This work was approved by the Ethics Committee of General Hospital of Northern Theater Command (EC-2020-KS-047).

### 2.2. Cell Culture

Human thyroid epithelial cell line Nthy-ori3-1 and human TC cell lines (TPC-1 and K-1) used in the study were all purchased from the American Type Culture Collection (ATCC; Manassas, VA, USA). Cells were cultured in DMEM supplemented with 10% fetal bovine serum (FBS) and 1% penicillin/streptomycin (Gibco, Grand Island, NY, USA). All the cell lines were confirmed to be free of mycoplasma contamination and cultured with 5% CO_2_ at 37°C.

### 2.3. Plasmid and Cell Transfection

miR-330-5p mimic, miR-330-5p inhibitor, and their negative controls (NC mimic and NC inhibitor) were chemically synthesized by GenePharma Company (Shanghai, China). FOXE1 plasmid was constructed from pcDNA3.1 vector, and the pc-NC served as negative control. Constructs were validated by DNA sequencing. All oligonucleotides and plasmids were transfected into cells with Lipofectamine 2000 (Invitrogen, CA, USA) when the cells had reached 80% confluency. Two days after transfection, the cells were collected for following use.

### 2.4. Cell Counting Kit-8 (CCK-8) Assay

We used a CCK-8 kit (Beyotime, Shanghai, China) according to the manufacturer's protocol to measure the capability of cell proliferation. In brief, TPC-1 and K-1 cells were seeded in 96-well plates and transfected with miR-330-5p inhibitor or NC inhibitor for 24, 48, and 72 h, and then 10 *μ*L CCK-8 assay solution was added to each well and incubated for 2 h at 37°C. The absorbance at 450 nm was detected using a microplate reader (Thermo Fisher Scientific, Waltham, MA, USA) to assess cell proliferation.

### 2.5. EdU Incorporation Assay

The EdU assay was conducted to assess the role of miR-330-5p in the proliferation of TPC-1 and K-1 cells. Cells were incubated in 96-well plates with miR-330-5p inhibitor or NC inhibitor transfection for 48 h. After that, EdU dissolved in medium was added to each well and then incubated for about 2 h at 37°C. Then the cells were fixed and stained with Hoechst33342 and Apollo reaction mixture. The proportion of cells that incorporated EdU was determined through fluorescence microscopy (Olympus, Tokyo, Japan).

### 2.6. Wound-Healing Assay

Wound-healing assay was used to investigate the role of miR-330-5p in TC cell lines metastasis. Briefly, TPC-1 and K-1 cells were seeded into six-well plates until they reached approximately 80% confluency. Then, a linear scratch was made with a 200 *μ*L pipette tip. After scratching, the floating cells were washed with PBS five times and then fresh medium was added. Scratch gaps were photographed at two time points (0 and 48 h) with a microscope (Olympus, Tokyo, Japan).

### 2.7. Transwell Assay

Transwell assay was used to investigate the role of miR-330-5p in TC cell migration and invasion. For Transwell migration assay, cells (1 × 10^5^) were suspended in 200 *μ*L serum-free medium and then added to the upper chambers of 8.0 *μ*m pores (Merck kGaA, Darmstadt, Germany). For Transwell invasion assay, cells (2 × 10^5^) were added to the upper chambers precoated with diluted Matrigel (1 : 5, BD Biosciences, USA) on their membrane. We used DMEM with 10% FBS to fill the lower chamber. Following incubation for 48 h, the nonmigrated/invaded cells on the upper surface of the membrane were removed, while migrated or invaded cells on the bottom surface were fixed with 4% paraformaldehyde and stained with 0.1% crystal violet for 30 min. After that, the cells were counted under a microscope (Olympus, Tokyo, Japan) in five randomly chosen microscopic fields.

### 2.8. Dual-Luciferase Reporter System Assay

For detecting the relationship between FOXE1 and miR-330-5p, we constructed the sequences containing the wild-type (WT) or mutant (Mut) 3′UTR of FOXE1. HEK 293T cells were cultured in a 24-well plate at a density of 70–90%, and then the cells were cotransfected with FOXE1-WT/FOXE1-Mut and miR-330-5p mimic or NC mimic using Lipofectamine 2000 (Invitrogen, CA, USA). About 48 h later, Renilla luciferase was measured in cell lysates using a Dual-Luciferase Reporter Assay System (Promega, Madison, WI, USA). Luciferase activity values were normalized relative to that of the Renilla luciferase internal control.

### 2.9. Quantitative Real-Time PCR (qRT-PCR) Assay

Total RNA was separated from different cells using TRIzol reagent (Invitrogen, CA, USA) following the manufacturer's protocol. The RNA (1 *μ*g) was reversely transcribed to cDNA with Prime Script RT Master Mix Kit (Takara, Otsu, Japan). qRT-PCR was performed by using SYBR Premix Ex Taq^TM^ (Takara, Otsu, Japan) on StepOnePlus™ Real-Time PCR System (Applied Biosystems, Thermo Fisher Scientific, USA). U6 or GAPDH was used as a normalized control. The sequences of primers are shown as follows: miR-330-5p, forward: 5′-TCTCTGGGCCTGTGTCTTAGGC-3′; reverse: 5′-CTAAGACACAGCCCAGAGATT-3′; U6 forward: 5′-CTCGCTTCGGCAGCACA-3′; reverse: 5′-AACGCTTCACGAATTTGCGT-3′; GAPDH, forward: 5′-ACCTGACCTGCCGTCTAGAA-3′; reverse: 5′-TCCACCACCTGTTGCTGTA-3′; FOXE1 forward: 5′-GCTGGTTTTCCCTGTCTCTG-3′, reverse: 5′- AGATGGGGGAGACTGAAGGT-3′.

### 2.10. Western Blot Analysis

Total protein was extracted from cells using lysis buffer and the protein concentration was quantified with the BCA method. The equal amounts of protein were separated by SDS-PAGE and then transferred onto PVDF membranes (Millipore, Billerica, USA). After blocking with 5% nonfat milk solution for 2 h, the membrane was then incubated with rabbit anti-FOXE1 antibody (1 : 1000, Abcam, Cambridge, MA, USA) overnight at 4°C and then with appropriate secondary antibodies (HRP-conjugated goat anti-rabbit IgG) at room temperature for 1 h. *β*-Actin was used as a loading control. The protein-antibody complexes were visualized by the Chemiluminescence ECL Kit (Millipore, Billerica, MA, USA).

### 2.11. Statistics

Data were represented as means ± standard deviation (SD) from at least three independent experiments. Student's *t*-test or one-way ANOVA followed by post hoc Bonferroni test was used to examine the differences between two groups or for multiple-group comparisons. All statistical tests were two-tailed. ^*∗*^*P* < 0.05 or ^#^*P* < 0.05 indicated statistical significance.

## 3. Results

### 3.1. miR-330-5p is Upregulated in TC Tissues and Cells

**Figure 1:**
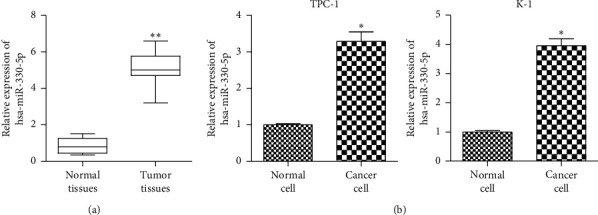
miR-330-5p is upregulated in TC tissues and cells. (a) qRT-PCR showed that miR-330-5p expression was upregulated in tumor tissues (*n* = 30) compared to normal tissues (*n* = 30). ^*∗∗*^*P* < 0.01*versus* normal tissue. (b) qRT-PCR tested the expression of miR-330-5p in TPC-1 and K-1 cells. Values are mean ± SD. ^*∗∗∗*^*P* < 0.005*versus* normal cells. *n* = 3 per group.

To identify the role of miR-330-5p in TC, the expression levels of miR-330-5p were analyzed in TC samples through qRT-PCR. The results revealed that miR-330-5p was significantly upregulated in human TC tissues compared with normal tissues (Figure 1(a)). In addition, the expression level of miR-330-5p was higher in TC cell lines (TPC-1 and K-1) than in normal cells (Figure 1(b)). These results revealed a positive correlation between high expression of miR-330-5p and poor prognosis of TC.

### 3.2. Knockdown of miR-330-5p Inhibits the Progression of TC

**Figure 2:**
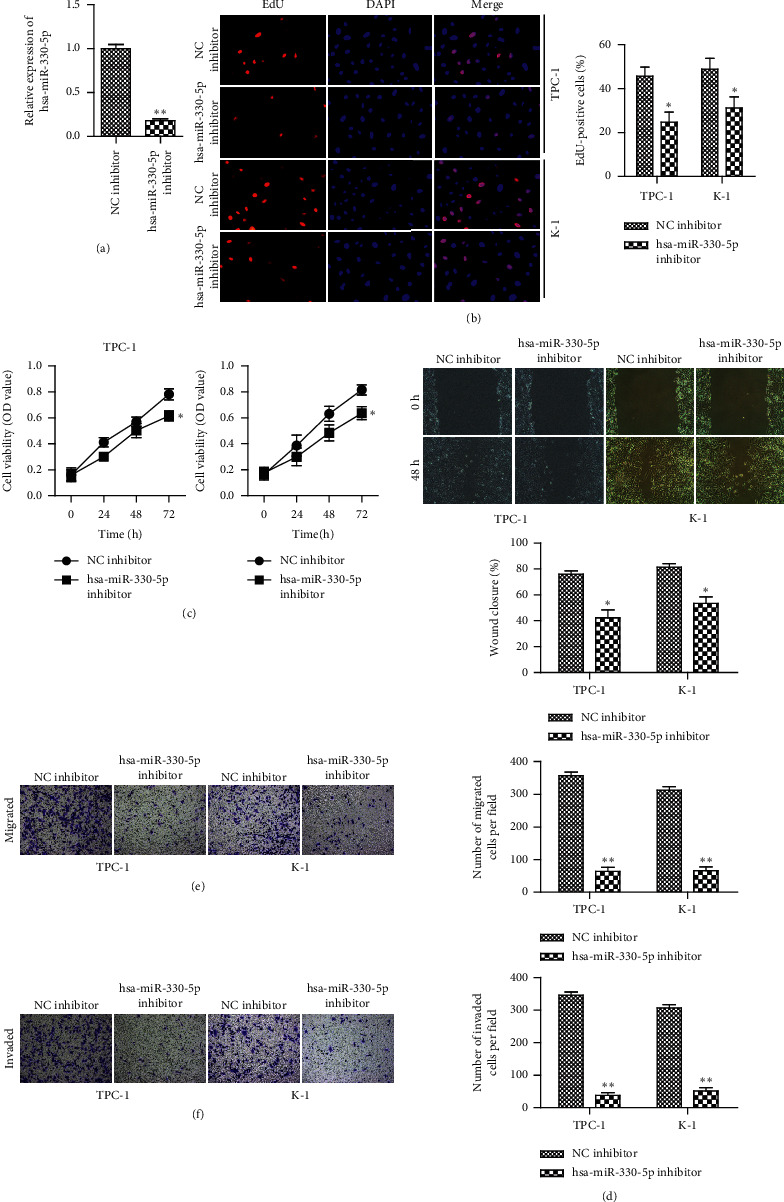
miR-330-5p promotes proliferation, migration, and invasion of TC. TPC-1 and K-1 cells were transfected with miR-330-5p inhibitor and its negative inhibitor (NC inhibitor). (a) qRT-PCR analysis showed that miR-330-5p was downregulated after transfecting with miR-330-5p inhibitor in TPC-1 and K-1 cells. (b) EdU and (c) CCK-8 assays showed that downregulated miR-330-5p inhibited cell proliferation in TPC-1 and K-1 cells. (d) Wound-healing and (e, f) Transwell assays for the migration and invasion abilities. Values are mean ± SD. ^*∗*^*P* < 0.05 and ^*∗∗*^*P* < 0.01*versus* NC inhibitor, with *n* = 3 per group.

In order to investigate the influence of miR-330-5p on TC progression, miR-330-5p inhibitor and its negative control (NC inhibitor) were transfected into TPC-1 and K-1 cells, respectively. qRT-PCR assay indicated that transfection with miR-330-5p inhibitor significantly reduced the expression of miR-330-5p (Figure 2(a)). The results of CCK-8 and EdU assay demonstrated that transfection with miR-330-5p inhibitor significantly decreased the proliferation of TPC-1 and K-1 cells (Figures 2(b) and 2(c)). It is well known that migration and invasion are the main characteristics of malignant tumors. Then we detected whether miR-330-5p was involved in the process of migration and invasion in TPC-1 and K-1 cells. Wound-healing and Transwell assays were performed to detect the migration and invasion abilities of TPC-1 and K-1 cells. The data showed that downregulation of miR-330-5p markedly decreased cell migration and invasion in TPC-1 and K-1 cells (Figures 2(d)–2(f)).

### 3.3. FOXE1 Is a Direct Target of miR-330-5p in TC

**Figure 3:**
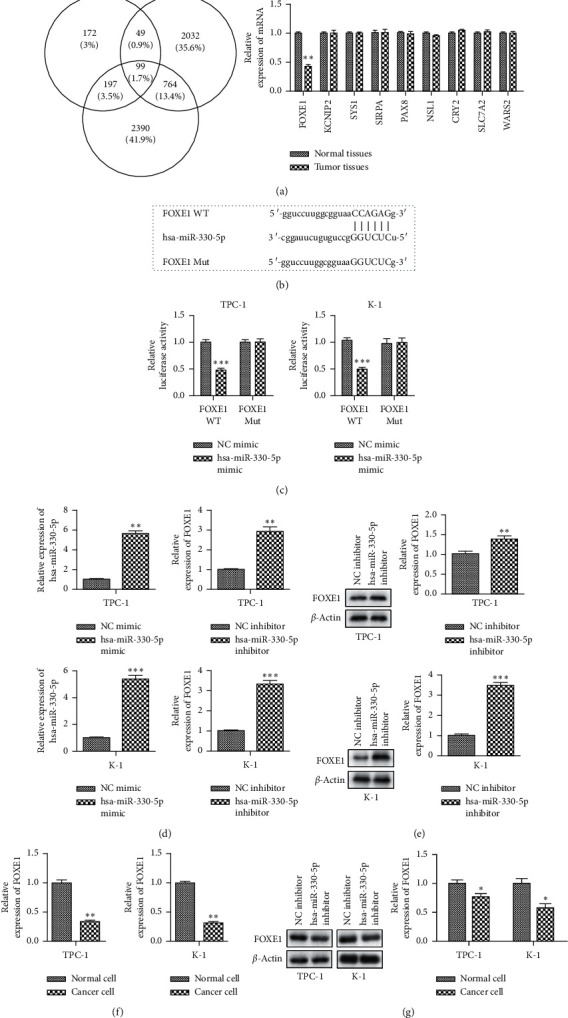
FOXE1 is a direct target of miR-330-5p in TC. (a) Schematic representation of the procedure for the identification of putative miR-330-5p target genes. As described in the Methods section, the analysis derives from an integration of miR-330-5p targets predicted by TargetScan, miRwalk, and ENCORI software. (b) Bioinformatic analysis of the predicted binding sites of FOXE1 and miR-330-5p. (c) Luciferase reporter assay detected the luciferase activity of reporter vector containing WT or mutant form of FOXE1 3′-UTR along with NC mimic or miR-330-5p mimic. (d) qRT-PCR and (e) western blot analysis showed that FOXE1 was upregulated in TPC-1 and K-1 cells with miR-330-5p inhibitor transfection. ^*∗∗*^*P* < 0.01*versus* NC inhibitor. (f-g) qRT-PCR and western blot assay showed that the expression levels of FOXE1 were decreased in TPC-1 and K-1 cells. Values are mean ± SD. ^*∗*^*P* < 0.05 and ^*∗∗*^*P* < 0.01*versus* normal cells.

Having determined the importance of miR-330-5p in regulating proliferation, migration, and invasion of TPC-1 and K-1 cells, we searched for critical direct targets that could explain miR-330-5p′s biological effects. Then we used three miRNA target prediction algorithms (TargetScan, miRwalk, and ENCORI) to search for genes that were directly regulated by miR-330-5p. Among all the targets, FOXE1 caught our attention (Figure 3(a)), since FOXE1 is a specific thyroid transcription factor and its suppressive effects in TC cells are well known [18]. TargetScan (http://www.targetscan.org) analysis predicted the binding site between miR-330-5p and FOXE1 (Figure 3(b)). To further validate the relationship between miR-330-5p and FOXE1, luciferase reporter assay was performed. To this end, we constructed luciferase reporter vector with the 3′ UTR of wild-type (WT) or mutant (Mut) FOXE1. We found that cotransfected miR-330-5p mimic and FOXE1-WT into HEK293 T cells significantly decreased the luciferase activity, while no significant alterations were observed in the luciferase activity of mutant FOXE1 (Figure 3(c)). Moreover, we found that FOXE1 was negatively regulated by miR-330-5p. The mRNA level of FOXE1 in TPC-1 and K-1 cells was markedly increased in miR-330-5p inhibitor group compared with control group (Figure 3(d)). qRT-PCR and western blot assays indicated that the expression level of FOXE1 was lower in cancer cells than in normal cells (Figures 3(e)–3(g)). These results demonstrated that FOXE1 is a direct target of miR-330-5p.

### 3.4. Overexpression of FOXE1 Reverses the Promotive Effects of miR-330-5p on the Progression of TC

**Figure 4:**
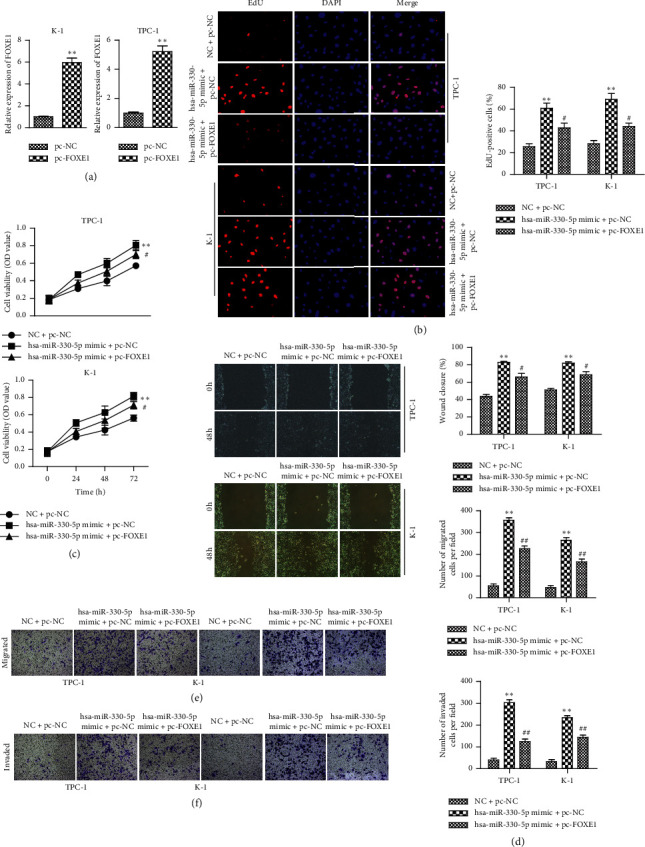
Restoration of FOXE1 reverses the effects of miR-330-5p on migration, invasion, and proliferation of TC. (a) qRT-PCR detection showed that FOXE1 levels were successfully enhanced by transfection with pc-FOXE1 in both TPC-1 and K-1 cells. (b) CCK-8 assays for the cell proliferation at the indicated time points (24, 48, and 72 h). (c) EdU assays for the cell proliferation. (d) Wound-healing and (e) Transwell assays for the migration and invasion abilities. (f) TPC-1 and K-1 cells were transfected with NC + pc-NC, miR-330-5p + pc-NC, or miR-330-5p inhibitor + pc-FOXE1. Values are mean ± SD. ^*∗∗*^*P* < 0.01*versus* NC + pc-NC. ^#^*P* < 0.05 and ^##^*P* < 0.01*versus* miR-330-5p mimic + pc-NC.

To confirm that miR-330-5p regulated proliferation, migration, and invasion of TC through targeting FOXE1, the vector pc-FOXE1 was first conducted to overexpress FOXE1. qRT-PCR was used to verify that FOXE1 level was successfully enhanced by transfection with pc-FOXE1 in both TPC-1 and K-1 cells (Figure 4(a)). Proliferative capacity was determined by CCK-8 and EdU assays. The results demonstrated that upregulation of miR-330-5p significantly enhanced the viabilities of TPC-1 and K-1 cells, while overexpression of FOXE1 reversed the effects of miR-330-5p mimic on cell proliferation (Figures 4(b) and 4(c)). The results of wound-healing and Transwell assays showed that FOXE1 overexpression reduced the activation of migration and invasion in TPC-1 and K-1 cells, which were caused by miR-330-5p overexpression (Figures 4(d)–4(f)). Our results indicated that FOXE1 is a functional mediator of miR-330-5p and plays a key role in TC proliferation, migration, and invasion.

## 4. Discussion

TC with higher morbidity and mortality is still a great challenge to search for novel therapeutic strategies. Numerous studies have demonstrated that dysregulation of miRNAs promotes the occurrence and development of tumors [19–21]. Therefore, further exploration of novel miRNAs may provide us with a better strategy for TC treatment. In this study, miR-330-5p was upregulated in TC tissues and cell lines. Moreover, downregulation of miR-330-5p significantly decreased cell proliferation, migration, and invasion. FOXE1 was first identified as a direct and functional target of miR-330-5p.

The development and progression of TC are related to various factors, among which miRNAs have great significance as a biomarker and molecular target for TC. Recently, the functional role of miR-330-5p in malignant tumors has been proposed. Xiao S *et al.* demonstrated that miR-330-5p targets SPRY2 to promote hepatocellular carcinoma progression via MAPK/ERK signaling [13]. Bibby et al. reported that silencing miR-330-5p increases MMP1 expression and promotes an invasive phenotype in esophageal adenocarcinoma [22]. It is also found that loss of miR-330-5p leads to PAX8 upregulation by LDR and suppression of thyroid carcinogenesis [23]. However, the biological functions and potential molecular mechanisms of miR-330-5p in the proliferation and metastasis of TC remain to be further elucidated. In our study, miR-330-5p was found to be highly expressed in TC tissues and cell lines. Functionally, it was determined that downregulation of miR-330-5p significantly inhibited tumor proliferation, migration, and invasion. These results suggested that miR-330-5p may act as an oncogenic factor of TC and abnormally elevated miR-330-5p may be a predictor of poor prognosis in TC patients.

Many studies have shown that miRNAs perform their functions through inhibiting the expression of target gene [24–26]. Our study first determined the tumor promoting effect of miR-330-5p in TC, but the underlying mechanism remains unclear. Therefore, we used three miRNA target prediction algorithms (TargetScan, miRwalk, and ENCORI) to predict the candidate target gene of miR-330-5p. Among the target genes, FOXE1 is a specific thyroid transcription factor. Therefore, our follow-up study was conducted on FOXE1. Studies have shown that FOXE1 plays an important role in the development, proliferation, and differentiation of thyroid cells [27, 28]. The results of our study were consistent with these previous reports. In this study, it is shown that FOXE1 was involved in the progression of TC. Dual-luciferase reporter assays confirmed that miR-330-5p directly bound to its 3′UTR and overexpression of miR-330-5p could suppress FOXE1 expression. Furthermore, FOXE1 expression is decreased in tumor tissues and TC cell lines; and FOXE1 overexpression partially rescued the effects of miR-330-5p on cell proliferation, migration, and invasion in TPC-1 and K-1 cells.

In conclusion, we first demonstrated the biological functions of miR-330-5p in TC. Downregulation of miR-330-5p expression significantly inhibited the proliferation, invasion, and metastasis of TC cells. As a direct target gene of miR-330-5p, FOXE1 reversed the effects of miR-330-5p on TC progression. Thus, miR-330-5p and FOXE1 may be used as new predictive indicators and therapeutic targets for TC treatment strategies.

## Data Availability

All the data used to support the findings of this study are included within the article.
